# Declining nitric oxide bioavailability in cardiovascular aging: mechanistic insights and emerging interventions

**DOI:** 10.20517/jca.2025.14

**Published:** 2025-12-07

**Authors:** Hriju Adhikari, Priyanka Patel, Namratha Javvaji, Mark J. Crabtree, Jillian N. Simon

**Affiliations:** 1 Aging + Cardiovascular Discovery Center, Lewis Katz School of Medicine at Temple University, Philadelphia, PA 19140, USA.; 2 School of Biosciences, Faculty of Health and Medical Sciences, University of Surrey, Guildford GU2 7XH, UK.

**Keywords:** Nitric oxide, vascular dysfunction, cardiovascular aging, oxidative stress, myocardial remodeling, lifestyle interventions, Sirtuin 1 (SIRT1)

## Abstract

Nitric oxide (NO) is essential for maintaining normal cardiovascular function, and accumulating evidence suggests that its diminished bioavailability contributes to endothelial dysfunction, vascular stiffening, and impaired cardiac performance - hallmarks of cardiovascular aging. This review posits that reduced NO bioavailability with age stems from impaired endothelial and neuronal NO synthase activity, increased oxidative stress, and metabolic shifts that drive cardiovascular decline. We further discuss emerging research which highlights potential interventions, including dietary nitrate supplementation, caloric restriction, and exercise, that may restore NO signaling and counteract age-related cardiovascular dysfunction. These findings underscore the growing recognition of NO as a key regulator of cardiovascular aging and a promising therapeutic target. Addressing NO-related deficits could open new avenues for preventing and treating age-associated cardiovascular diseases, reshaping strategies for promoting healthy aging and longevity.

## Graphical Abstract

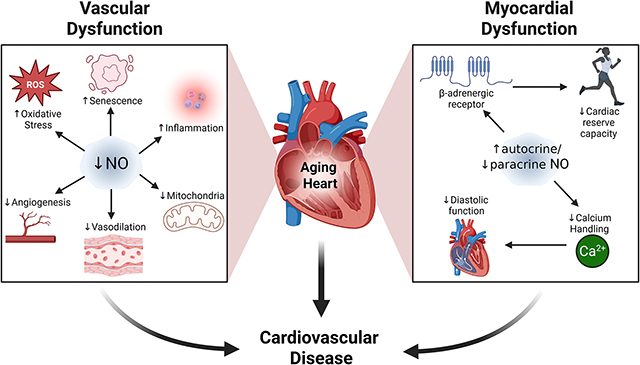

## INTRODUCTION

Cardiovascular aging is a leading contributor to morbidity and mortality worldwide, driven by progressive impairments in endothelial function, vascular compliance, and myocardial performance that emerge with advancing age^[1,2]^. Over the past few decades, significant strides have been made in understanding the cellular and molecular alterations associated with aging, such as cellular senescence, mitochondrial dysfunction, and defects in intracellular signaling. Despite these advances, age-related cardiovascular morbidities persist, underscoring the need for deeper insight into the underlying mechanisms driving cardiovascular aging.

Nitric oxide (NO) signaling plays a pivotal role in regulating cardiovascular function, and its dysregulation is central to both vascular and myocardial aging. Initially recognized for its vasodilatory effects in the endothelium, NO is essential for maintaining vascular tone, and its age-related decline contributes to vascular dysfunction, characterized by impaired vasodilation, increased oxidative stress, and endothelial cell senescence. Beyond the endothelium, NO is now also known to influence a wide array of processes in cardiomyocytes, including excitation-contraction (EC) coupling^[3–5]^, β-adrenergic responsiveness^[6]^, mitochondrial function^[7]^, and extracellular matrix remodeling^[8]^. However, its role in age-associated myocardial dysfunction remains less well understood. Studies over the past two decades have increasingly implicated dysregulated NO signaling - resulting from alterations in nitric oxide synthase (NOS) expression, cofactor availability, and oxidative stress - as a key mechanism underlying both vascular and myocardial dysfunction. However, our mechanistic understanding of how distinct NOS isoforms contribute to age-related alterations in NO bioavailability and signaling remains incomplete. As such, defining the precise role of NO in cardiovascular aging has become an area of active investigation, with growing recognition that restoring NO signaling may hold promise for mitigating age-related cardiac decline and improving outcomes in elderly patients with cardiovascular disease.

This review highlights the critical need to understand how aging disrupts NO production and signaling in both the endothelium and myocardium, and how these disruptions converge to drive vascular and myocardial dysfunction. While much of the foundational work in the field has focused on endothelial-derived NO and its role in vascular tone and systemic hemodynamics, comparatively less attention has been given to how NO signaling is altered within the aging myocardium itself. Addressing this gap is essential, as the heart is both a target and a modulator of nitroso-redox signaling. In this review, we examine how aging disrupts NO production and signaling in both the vasculature and myocardium, emphasizing the multifaceted consequences of NO deficiency - from endothelial dysfunction and oxidative stress to impaired myocardial relaxation and cardiac reserve. We aim to move beyond traditional paradigms by integrating mechanistic and translational insights that position NO not only as a marker of cardiovascular aging but as a causal driver of cellular senescence, dysfunction, and vulnerability to disease. In ng so, we hope to reframe NO signaling as a central, modifiable axis in age-related cardiovascular decline and to highlight promising directions for future investigation and therapeutic intervention.

## NO SYNTHESIS AND REGULATION

**Figure 1.**
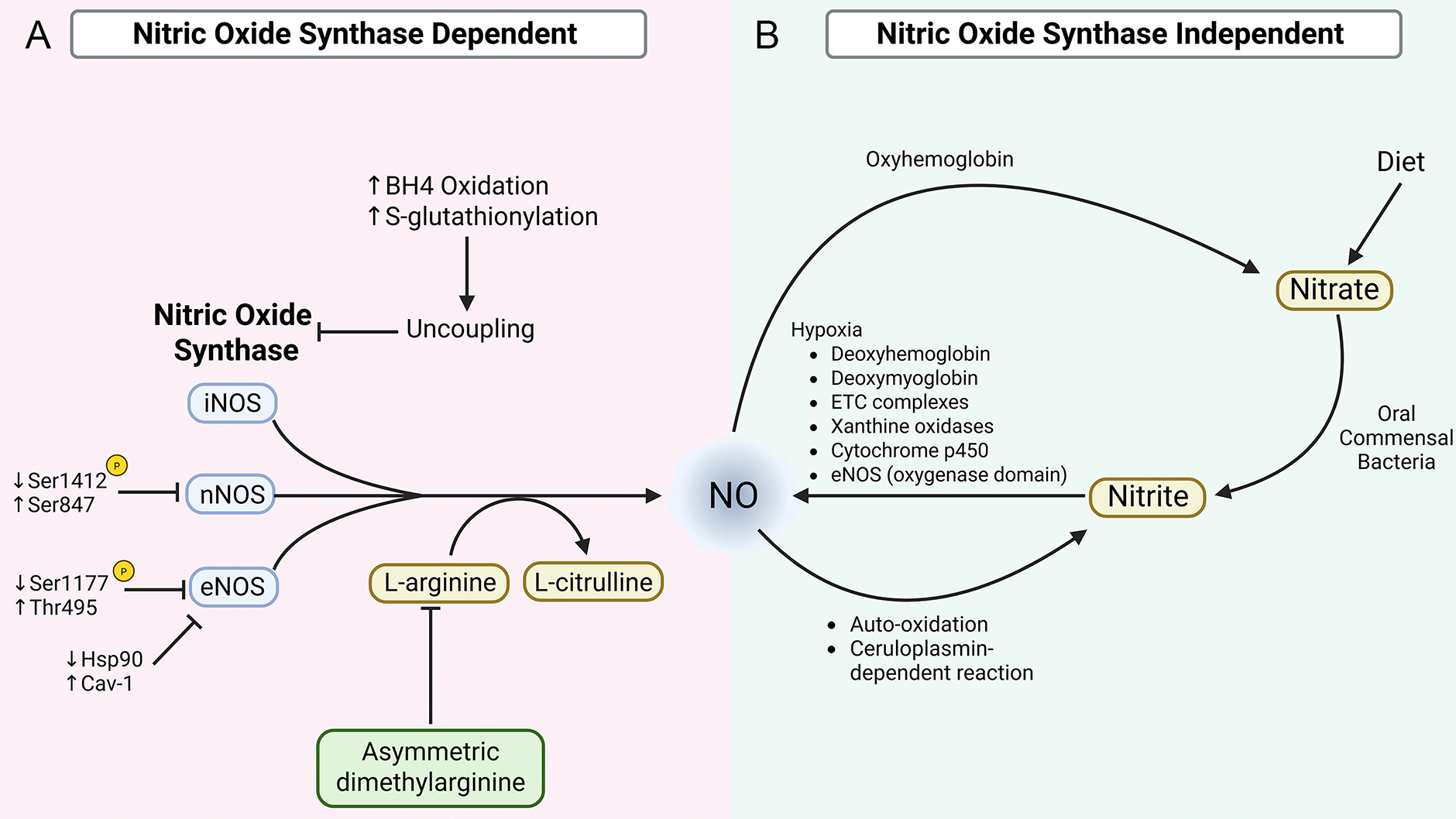
Synthesis and Regulation of NO. [(A) NOS-dependent NO production] NO is enzymatically synthesized through the conversion of L-arginine to L-citrulline by iNOS, nNOS, and eNOS. Each of these NOS isoforms is regulated by distinct phosphorylation sites, with phosphorylation of Ser1412 and dephosphorylation at Ser847 driving nNOS activity, and phosphorylation at Ser1177 and dephosphorylation at Thr495 driving eNOS activity. Age-related alterations of these phosphorylation states (as represented by the indicated arrows) reduce NOS activity in the cardiovascular system. Additionally, age-associated inhibitory NOS modulation by increased ADMA reduces L-arginine availability, further impairing NOS-mediated NO production. Moreover, age-associated depletion of BH_4_ levels and increased S-glutathionylation promote NOS uncoupling and further downregulate NO bioavailability. [(B) NOS-independent NO production] Alternatively, NO can also be produced by the reduction of nitrate and nitrite, derived from dietary sources or from the oxidation of NOS-derived NO. Circulating nitrate and nitrite act as storage forms of NO and can be reduced back to NO under hypoxic conditions. Reduction of nitrate/nitrite to NO is mediated by mechanisms involving deoxyhemoglobin, deoxymyoglobin, mitochondrial ETC complexes, xanthine oxidases, cytochrome 450, and the oxygenase domain of eNOS. Created in BioRender. Patel, P. (2025) https://BioRender.com/v5hq0f8.

In the healthy cardiovascular system, NO is synthesized through two distinct but complementary pathways that collectively regulate vascular tone, myocardial function, and cellular homeostasis. The primary source of NO is the NOS-dependent pathway, in which NOS enzymes catalyze the oxidation of L-arginine to produce NO and L-citrulline. In parallel, a NOS-independent pathway involving the sequential reduction of dietary or endogenous nitrate to nitrite and then NO serves as an alternative source, particularly under hypoxic or acidic conditions. Together, these pathways ensure tight spatial and temporal control of NO availability and signaling under physiological conditions. This section outlines the key mechanisms governing NO production and regulation in the unaged cardiovascular system, providing a foundation for understanding how these processes become dysregulated with advancing age [Figure 1].

### NOS pathway and regulation

Three distinct isoforms of NOS exist in mammals - neuronal NOS (nNOS or NOS1), inducible NOS (iNOS or NOS2), and endothelial NOS (eNOS or NOS3) - each encoded by a separate gene. Despite their names, both nNOS and eNOS are constitutively expressed in the cardiovascular system, where they generate NO in a calcium-dependent manner^[9,10]^. In contrast, iNOS expression is negligible in a healthy cardiovascular system but can be transcriptionally activated in response to injury or inflammation^[11–13]^.

All NOS isoforms function as homodimers, with each monomer consisting of an N-terminal oxygenase domain and a C-terminal reductase domain, linked together by a calmodulin (CaM)-binding sequence. These homodimers are bound and stabilized by heme, tetrahydrobiopterin (BH_4_), L-arginine, and zinc at the oxygenase domain^[14–17]^. When dimerized, electron transfer that drives NO synthesis begins at the reductase domain, where flavin cofactors (e.g., flavin adenine dinucleotide (FAD) and flavin mononucleotide (FMN)) and nicotinamide adenine dinucleotide phosphate (NADPH) initiate electron transfer to CaM^[18,19]^. From CaM, electrons are transferred to heme iron in the oxygenase domain, enabling the conversion of L-arginine and oxygen to L-citrulline and NO. Conversely, iNOS possesses a more tightly bound CaM, rendering it active upon expression, even at low calcium concentration^[20]^

In addition to dimerization, NOS activity and functionality are regulated by post-translational modifications and distinct intracellular localization. For instance, eNOS localizes to the plasma membrane caveolae and Golgi membrane upon myristoylation and palmitoylation of cysteine residues 15 and 26^[21]^. When eNOS is at the caveolae or the Golgi, phosphorylation of Ser1177 is required for activation and subsequent NO production^[22]^. Indeed, a critical mechanism underlying reduced NO bioavailability in vascular aging is the dysregulation of eNOS activity via inhibitory phosphorylation at Thr495. Phosphorylation at this residue stabilizes eNOS in an inactive conformation by sterically hindering CaM binding, a prerequisite for enzymatic activation^[23]^. While evidence of nNOS phosphorylation in cardiomyocytes remains limited, studies in neurons indicate that nNOS may also undergo inhibitory phosphorylation at Ser847 by calmodulin-dependent protein kinase I and II^[24]^, whereas phosphorylation at Ser1412 by protein kinase B (also known as AKT serine/threonine kinase 1) enhances its activity^[25]^. Similar to eNOS and nNOS, tyrosine residues on iNOS can also be phosphorylated to increase its activity^[26]^.

Furthermore, the allosteric binding of proteins to the NOS enzyme can also modulate functionality. At caveolae, the oxygenase domain of tyrosine-phosphorylated eNOS allosterically binds caveolin-1 (Cav-1)^[27,28]^. This interaction prevents the binding of CaM to eNOS, thereby inhibiting eNOS activity^[29]^. Both nNOS and eNOS are also inhibited by caveolin-3 (Cav-3)^[30]^. Conversely, heat-shock protein 90 (Hsp90), a chaperone protein that assists with protein folding and maintains protein stability, binds to eNOS and nNOS directly in a CaM-dependent manner^[31,32]^. This interaction releases NOS from Cav-1, thus allowing for enzyme activation. The binding of Hsp90 to both nNOS and eNOS also facilitates “re”-coupling of the enzyme and reduces superoxide (O_2_^−^) production^[33]^.

Substrate/cofactor availability is further required for NOS activity. Among the NOS cofactors, BH_4_ plays an essential role in enabling electron transfer to promote L-arginine oxidation, NO formation, and release^[34]^, in addition to its role as a homodimer stabilizer. During this reaction, electrons donated by NADPH at the carboxy-terminal reductase domain of NOS are passed to the heme catalytic center of the oxidase domain, where activation of molecular oxygen is “coupled” to NO synthesis by oxidation of L-arginine. The cofactor BH_4_ is required for these reactions; in its absence, electron flow to molecular oxygen becomes “uncoupled” from L-arginine oxidation, resulting in the production of O_2_^−^ instead of NO^[35,36]^. Oxidation of BH_4_ to BH_2_ can also facilitate uncoupling, as BH_2_ competes with BH_4_ for binding to NOS^[37]^. Unlike BH_4_, BH_2_ lacks cofactor activity, and thus, when BH_2_ binds to NOS, it disrupts electron transfer, causing the enzyme to uncouple and produce O_2_^−^. The combination of increased oxidative stress and impaired NO signaling resulting from NOS uncoupling has been implicated in the pathogenesis of a wide range of disease states, including atherosclerosis^[38]^, hypertension^[39–41]^, diabetes^[42,43]^ and more recently aging^[44,45]^. Additionally, post-translational modification of NOS by S-glutathionylation of cysteine residues in the reductase domain can also enable NOS uncoupling^[46]^.

### Nitrate-nitrite-NO

Historically, nitrate and nitrite were considered biologically inert molecules; however, extensive research over the past few decades has revealed that nitrate and nitrite serve as reserve pools of NO. Nitrate and nitrite are supplied to the body in two ways: (1) by the oxidation of endogenous NO produced by the NOS pathway; and (2) by consumption of vegetable diets rich in nitrate/nitrite. Evidence from pharmacological and genetic inhibition of eNOS demonstrating reduced nitrite level and elevated vascular resistance indicates that plasma nitrite concentration reflects NOS activity^[47]^. In fact, as much as 70% of nitrite is derived from NOS-dependent endogenous NO^[47]^. Under basal conditions, NO in the plasma reacts with oxyhemoglobin to produce methemoglobin and nitrate. NO also auto-oxidizes or undergoes ceruloplasmin-dependent reactions with copper to form nitrite^[48]^.

Beyond endogenous production, dietary intake provides an additional source of nitrate, which undergoes sequential reduction in the body. Dietary nitrate is reduced to nitrite by commensal bacteria in the oral cavity^[49]^. Nitrite is then absorbed into the circulation, adding to the reserve pool in the plasma and tissues alongside NOS-dependent nitrite^[48]^.

Nitrite is also readily reduced to NO under acidic and hypoxic conditions^[50]^. Rat hearts subjected to 30 min of myocardial ischemia demonstrated a significant conversion of isotopically labeled nitrite to NO^[50]^. Moreover, deoxyhemoglobin and deoxymyoglobin, which are increased in hypoxic conditions, have also been demonstrated to reduce nitrite to NO to drive hypoxic vasodilation^[51,52]^. Interestingly, this conversion is most robust in tissues with high mitochondrial activity, such as the liver and the heart^[53]^. Moreover, nitrite to NO conversion correlates with mitochondrial oxidative phosphorylation capacity, as inhibition of mitochondrial electron transport chain (ETC) complexes by rotenone, antimycin A, and myxothiazol suppressed NO formation under hypoxic conditions^[53]^. Alternatively, this finding could also suggest that ETC complexes are directly involved in nitrite reduction^[54]^.

Several other mammalian enzymes involved in nitrite reduction have been identified. Among these are xanthine oxidoreductases^[55,56]^, cytochrome p450 enzymes^[53]^, and eNOS. Most notably, under hypoxic conditions, eNOS does not convert L-arginine to NO^[57]^, as there is little oxygen available. Rather, the oxygenase domain of eNOS serves as a location for nitrite reduction^[57,58]^ and maintains NO levels to sustain vasodilation.

## NO AND VASCULAR AGING

In the late 1970s and 1980s, a series of groundbreaking studies identified that NO stimulated soluble guanylyl cyclase (sGC) to produce cyclic guanosine 3′,5′-monophosphate (cGMP), subsequently activating protein kinase G (PKG) to induce smooth muscle relaxation and vasodilation^[59,60]^. Following this discovery, NO quickly became recognized as a critical signaling molecule in the vasculature, playing a key role in maintaining endothelial function, vascular tone, and overall cardiovascular health. The age-related decline of vascular health, coined “vascular aging”, is characterized by increases in arterial stiffness, endothelial dysfunction, intima thickening, and chronic inflammation, all contributing to the pathogenesis of cardiovascular disease^[61]^. Given NO’s central role in vascular homeostasis, researchers began investigating whether age-related vascular dysfunction might be driven, at least in part, by alterations in NO signaling.

### Changes in NO bioavailability in vascular aging

Many studies have directly measured NO bioavailability in aged models and have obtained mixed results, with some studies reporting elevated circulating NO levels^[62–64]^, while others report reductions^[65–67]^. However, this can be explained given the dynamic and multifaceted roles of NO, as evaluating circulating NO levels may not be sufficient to fully understand the role of NO in aging: NO can either be released as a signaling agent through its interaction with thiol groups or be stored in tissues as nitrate/nitrite^[68,69]^. Despite evidence that nitrate/nitrite metabolism is maintained with age in rats, a recent study in young and old rats found that plasma and tissue nitrate levels increased with age, whereas nitrite levels decreased^[70]^. These findings suggest that nitrate and nitrite levels may be regulated independently, potentially via distinct pathways, and can vary with age and across different tissues.

#### Metabolites

Metabolites that can augment NO production, such as L-arginine, or inhibit NOS (e.g., asymmetric dimethylarginine (ADMA)) are reported to change with age. Serum collected from aging rats had a 70% decrease in L-arginine^[71]^ and a ~47% increase in ADMA^[72]^. Likewise, healthy men, but not women, showed an increase in ADMA with age, which was negatively correlated with flow-mediated dilation in the popliteal artery^[73]^. L-arginine levels in women were shown to change throughout menopause, with a statistically nonsignificant decrease during pre-menopause and an increase post-menopause^[74]^. More studies that examine NO reservoirs and associated metabolites may lend more insight into NO bioavailability changes that may occur during aging and explain the differences observed in levels of NO bioavailability.

#### NOS expression and activity

Variations in NOS expression have also been observed with aging and may contribute to the differences in NO bioavailability reported. As with circulating NO, studies on age-related changes in NOS expression and activity have yielded inconsistent results. In the aortas of aged rats, iNOS expression and activity rise, whilst eNOS expression is elevated, yet activity is decreased^[75,76]^. Another study found reduced Ser1177 phosphorylation and elevated phospho-Thr495 levels, supporting the finding of decreased eNOS activity observed in aortic endothelium from aged rats^[77]^. Contrary to these studies, van der Loo *et al.* (2000) found increased eNOS expression and NOS activity in aged rat aortas^[67]^, although the levels of BH_4_ and its oxidation status were not measured. In humans, aging has been associated with increased eNOS Ser1177 phosphorylation, indicating increased eNOS activity^[78]^, although the balance between activating (Ser1177) and inhibitory (Thr495) phosphorylation sites was not determined. Aged human umbilical vein endothelial cells (HUVECs), however, do exhibit a 6-fold increase in Thr495 phosphorylation alongside reduced Ser1177 phosphorylation, directly impairing NO synthesis^[79,80]^. This imbalance arises from age-related upregulation of Rho-kinase (ROCK), which directly phosphorylates Thr495, and from diminished protein kinase B (Akt/PKB) signaling responsible for Ser1177 activation^[80]^. Co-morbidities, such as hypertension, may further influence age-associated changes in NOS isoform expression and activity. Hypertension in aging rats shifts eNOS and iNOS expression by decreasing eNOS levels and raising iNOS^[81]^. Furthermore, Sirtuin 1 (SIRT1), a NAD^+^-dependent deacetylase, enhances eNOS activity by deacetylating and upregulating its expression. As NAD^+^ levels decline with age, SIRT1 expression and activity decrease, resulting in a decrease in eNOS activity^[82–85]^. Studies have demonstrated that activation of SIRT1 reduces vascular aging in aged mice^[86–88]^. Thus, despite extensive study, the full complexity of age-related NOS regulation remains unresolved, and investigations into more nuanced, context-specific changes may offer deeper insight. Moreover, observed differences in NOS expression may reflect species-specific variability or compensatory responses aimed at preserving NO bioavailability - hypotheses that warrant further investigation.

#### NOS uncoupling

In addition to changes in the expression of NOS, NO bioavailability becomes attenuated by the uncoupling of electron flow from arginine oxidation in eNOS. This mechanism results in a decreased production of NO and instead generates O_2_^−[89]^. Studies have examined age-induced eNOS uncoupling and have reported elevations in BH_2_ and diminished BH4 levels and significant O_2_^−^ production^[90–92]^. Similarly, in the mesenteric arteries of aged mice, the eNOS monomer/dimer ratio was elevated in old mice, and N(G)-nitro-L-arginine methyl ester (L-NAME) inhibitable O_2_^−^ production was elevated^[91]^, findings that were replicated in the aortas of aged rats^[93,94]^. Importantly, in all these studies of eNOS uncoupling in aged animal models, O_2_^−^ levels and vascular function were restored following supplementation with either BH_4_ or its precursor, sepiapterin, demonstrating the importance of BH_4_ and maintenance of eNOS coupling in the vasculature. Moreover, a reduction in the binding of eNOS to Hsp90, which facilitates the coupling of eNOS, has been observed in aged piglets^[95]^. Additionally, Cav-1 expression is increased in aged endothelial cells, resulting in the sequestration of eNOS within the caveolae^[32]^. Together, the reduction in Hsp90 and the increase in Cav-1 further exacerbate the uncoupling and decrease the activity of eNOS. The age-related uncoupling of eNOS can lead to an increase in O_2_^−^, which can react with NO to form peroxynitrite^[96]^. This new free radical can then react with tyrosine residues in proteins to produce 3-nitrotyrosine, a marker of nitrosative stress^[96]^. Consistent with this idea, an increase in total protein nitrotyrosine is observed in the vasculature of aged mice^[91]^. Specifically, an increase in nitrotyrosine and eNOS colocalization in aged mesenteric arteries was observed. This post-translational modification of eNOS by nitrotyrosine not only impairs its enzymatic activity but also perpetuates a deleterious cycle of reduced NO production and increased oxidative stress in the aging vasculature.

#### NOS cofactor availability

Changes in NOS cofactor availability with age may also indirectly affect NO bioavailability. Arginase isoforms (Arg-I & II) convert L-arginine into L-ornithine as part of the urea cycle to dispose of ammonia^[97]^. This enzyme is an important regulator of NO production as it competes with NOS for L-arginine. As Arginase expression or activity increases, endothelial dysfunction also rises, and NO bioavailability decreases^[98]^. This relationship between arginase activity, endothelial dysfunction, and NO bioavailability mirrors the patterns observed in vascular aging^[99]^. Despite these parallels, investigations into arginase expression and activity in aged tissues have yielded conflicting findings, where some studies show that Arginase activity and expression rise in aged animals^[100–102]^, while others observe no difference^[103,104]^ or have reported a decrease^[105,106]^. Together, these findings underscore the multifactorial regulation of NO signaling in the aging vasculature, involving alterations in substrate/cofactor availability, enzyme expression and activity, and redox balance.

#### Sex-specific differences in NO signaling and vascular aging

Sex-specific differences in NO signaling emerge as a critical determinant of vascular aging patterns between men and women, with estrogen and testosterone exerting distinct modulatory effects on NO bioavailability and cardiovascular health. Premenopausal women demonstrate superior vascular protection through estrogen-mediated enhancement of eNOS expression and activity, resulting in greater NO synthesis, improved endothelium-dependent vasodilation, and lower arterial blood pressure compared to age-matched men^[107–109]^. This protective effect involves multiple mechanisms including estrogen receptor-α activation that upregulates eNOS gene expression, enhanced phosphorylation at activating sites (Ser1177), and preservation of BH_4_ cofactor availability, collectively maintaining eNOS coupling and preventing O_2_^−^ production^[109,110]^. The menopause transition marks a critical inflection point where women experience an accelerated decline in endothelial function that surpasses the gradual deterioration observed in men, coinciding with estrogen deficiency-induced reductions in NO bioavailability, increased oxidative stress, and loss of anti-inflammatory protection^[109,111,112]^. Conversely, testosterone’s role in male vascular aging presents a more complex picture, with physiological concentrations promoting NO synthesis through androgen receptor-mediated activation of extracellular signal-regulated protein kinase (ERK)1/2 and phosphatidylinositol 3 kinase (PI3K)/Akt pathways^[113]^, yet age-related testosterone decline potentially contributes to endothelial dysfunction through increased endothelin-1 signaling and reduced antioxidant capacity^[111,113,114]^. The differential reliance on basal NO bioavailability also exhibits sex-specific patterns, with females demonstrating greater dependence on endogenous NO production while males show enhanced responsiveness to exogenous NO supplementation, suggesting fundamental differences in vascular NO regulation that may inform targeted therapeutic strategies for age-related cardiovascular dysfunction^[115]^. These sex-specific differences in NO signaling underscore the importance of personalized approaches to cardiovascular aging interventions, recognizing that optimal strategies for preserving vascular health may vary significantly between men and women across different life stages.

### Impact of changes in NO bioavailability on vascular aging

#### Vasodilation

Although reported changes in NO bioavailability in aged animals have been inconsistent, there is a clearer consensus regarding the functional consequences of NO signaling on vascular function with age. In fact, a decrease in NO-dependent endothelial vasodilation is now considered a hallmark characteristic of aging vasculature. Studies using vascular rings (aortic or mesenteric) from older animals consistently demonstrate reduced relaxation responses to NO donors compared to those from young animals^[116,117]^. Furthermore, acetylcholine-induced vasodilation, which is inhibited by NOS blockers in young animals and humans, becomes NOS-independent or is abolished altogether in older counterparts^[117,118]^. Supporting this, BH_4_ infusion has been shown to restore vasodilation in healthy older men by enhancing NO production^[119]^. Interestingly, vascular function appears better preserved in aging females^[120,121]^. Overall, these studies have largely shown that aging is associated with a decline in NO-mediated vascular responsiveness.

#### Oxidative stress

Another NO-driven hallmark of aging is the increase in oxidative and nitrosative stress^[122,123]^. With age, NO is scavenged by reactive oxygen species (ROS), specifically O_2_^−^. NO reacts rapidly with O_2_^−^ to form peroxynitrite^[96]^. This new free radical can then react with tyrosine residues in proteins to produce 3-nitrotyrosine, a marker of nitrosative stress. For example, the peroxynitrite nitration of manganese O_2_^−^ dismutase (MnSOD) inhibits its function as a ROS scavenger and further exacerbates oxidative stress^[67]^. Changes in NO bioavailability in turn may also be influenced by the rise in ROS. ROS promotes eNOS uncoupling, further exacerbating the reduced bioavailability of NO^[124]^. A study found that aortic tissue isolated from aged rats had elevated O_2_^−^production and 3-nitrotyrosine modifications compared to the young rats^[67]^. This indicates that an overall increase in oxidative stress may influence the loss of NO bioavailability with aging. Increased oxidative stress accelerates vascular aging by inducing molecular and pathological changes. One significant consequence is the deterioration of arterial structural integrity with aging^[125]^. Conversely, anti-oxidative treatments have been shown to mitigate these effects, helping to prevent large artery stiffening and preserve vascular function^[126]^.

#### Metabolism

The functional integrity of the heart and vasculature is dependent on normal cellular metabolism. A growing body of research highlights the impact of NO on energy metabolism in vascular aging through mitochondria. Mitochondria are critical organelles involved in energy metabolism, and their decline in function has been associated with aging. NO can impair mitochondrial function both directly, through interactions with the energy transport chain, and indirectly, through increased nitrosative and oxidative stress. NO can directly impact the ETC by either binding, nitration, or s-nitrosylation of various complexes, impairing their function and inhibiting mitochondrial respiration^[127]^. As the efficacy of the ETC decreases, electron leakage increases, resulting in elevated ROS. The proximity of ROS to mitochondrial DNA (mtDNA) increases mtDNA mutations, which further accelerates mitochondrial decline. Mitochondrial oxidative stress is a key characteristic of aging that promotes vascular dysfunction^[128–131]^. Studies have shown that treatment with mitochondrial-targeted antioxidants, such as mitoquinol mesylate (MitoQ), resveratrol, and SS-31 (d-Arg-2′,6′-dimethyltyrosine-Lys-Phe-NH_2_), has improved endothelial function in aged arteries of rodents^[87,130,132]^. Furthermore, increased peroxynitrite availability promotes post-translational modifications, such as tyrosine nitration, altering the structure and function of critical mitochondrial proteins^[127,133,134]^. NO promotes mitochondrial fission by nitrosating Parkin, which upregulates dynamin-related protein 1 (Drp1)^[135]^, the primary driver of mitochondrial fission. NO also increases Drp1 phosphorylation at Ser616, further activating mitochondrial fission^[135]^. Increased S-nitrosated Drp1 is often observed in aged cells and is associated with impaired energy metabolism^[136]^. Overall, impaired NO signaling in aging disrupts mitochondrial bioenergetics and function, further exacerbating vascular dysfunction.

#### Cellular senescence

Cellular senescence, the cessation of cell proliferation after a cell has reached its maximum division potential, is a natural component of aging. Studies accelerating senescence in aging models have shown a progression of heart failure and endothelial dysfunction^[137–139]^. Moreover, chronic senolytic treatment improved endothelial dysfunction in aged mice^[140]^. NO has been shown to regulate cellular senescence by enhancing telomerase activity, which is essential for maintaining chromosomal stability and prolonging cell lifespan^[141]^. In HUVECs, treatment with an NO donor decreased the proportion of senescent cells, whereas inhibition with L-NAME increased the proportion of senescent cells^[141]^. This suggests that NO plays a protective role against endothelial senescence and vascular aging. Furthermore, the expression of SIRT1 in senescent cells is also reduced, which subsequently leads to decreased activity of eNOS and further diminishes the protective effects of NO^[83]^.

#### Inflammation

Aging is associated with chronic low-grade inflammation, often referred to as “inflammaging”^[142]^. Studies have shown that aging is correlated with a shift in gene expression favoring pro-inflammatory cytokines^[131,143,144]^. The consequences of chronic inflammation on vascular dysfunction and formation of atherosclerotic plaques are well-studied. NO can exert anti-inflammatory effects in the vasculature by inhibiting leukocyte adhesion and vascular smooth muscle cell (VSMC) proliferation^[145,146]^. However, with aging, reduced NO bioavailability diminishes these protective effects and exacerbates vascular inflammation. Moreover, NO-dependent elevated ROS levels activate nuclear factor kappa B (NF-κB), which promotes the expression of pro-inflammatory mediators. Both endothelial cells and VSMCs exhibit increased NF-κB activity with aging^[86,147]^. Additionally, in aged rat arterioles, an increase in expression of iNOS and formation of peroxynitrite is observed^[148]^. The increase in peroxynitrite promotes vascular dysfunction in addition to iNOS-induced regulation of inflammatory cytokines, such as tumor necrosis factor-alpha (TNFα). Inhibition of NO-induced apoptosis in coronary arteries by activating TNFα; however, treatment of aged arteries with NO donor or TNFα inhibitor decreased this apoptosis^[149]^.

#### Angiogenesis

Angiogenesis is an essential physiological process for tissue repair and homeostasis. However, aberrations in angiogenesis contribute to a variety of age-associated pathologies, including stroke, myocardial infarction, and cancer. Aging is associated with a decline in angiogenic capacity, which has been linked to reduced vascular NO bioavailability^[150]^. Multiple studies have demonstrated that various NO delivery methods enhance angiogenesis in animal models^[151–154]^. NO influences angiogenesis through several mechanisms, including endothelial cell proliferation, tip cell formation, and migration. One study found that NO enhances endothelial cell proliferation by modulating DNA synthesis. This effect was abolished following pretreatment with the NO inhibitor, L-NAME^[155]^. NO modulates the formation of tip cells via the regulation of the sGC pathway. In human endothelial cells, L-NAME decreases tip cell formation, whereas the presence of an NO donor increases tip cell formation^[156]^. Furthermore, NO has been shown to enhance endothelial cell migration, another essential step in angiogenesis. Studies using HUVECs demonstrated increased cell migration upon treatment with NO donors. Similarly, in ex vivo models, NO donors promoted angiogenesis in rat aortic rings, further emphasizing the pro-angiogenic properties of NO^[157]^. Moreover, NO can control angiogenesis through the vascular endothelial growth factor (VEGF) signaling pathway. NO activates hypoxia-inducible factor 1-alpha (HIF-1α), which upregulates VEGF expression^[158]^. The addition of NO donors or cofactors increases VEGF synthesis, while inhibition of NO production via L-NAME decreases VEGF synthesis in VSMCs^[159]^. Moreover, VEGF-induced endothelial cell proliferation is attenuated by L-NAME^[160]^. NO is a critical regulator of angiogenesis, and thus, the limited bioavailability with age greatly impacts the vascular angiogenic capacity.

Changes in NO bioavailability are a critical mechanism of vascular aging, contributing to endothelial dysfunction, inflammation, arterial stiffening, and culminating in the progression of atherosclerosis. Over time, atherosclerosis and endothelial dysfunction will increase pulse pressure and cardiac afterload, compounding the risk of cardiovascular diseases. Collectively, NO-related changes drive an atherosclerotic phenotype in aging, resulting in an increased risk for cardiovascular disease.

**Figure 2.**
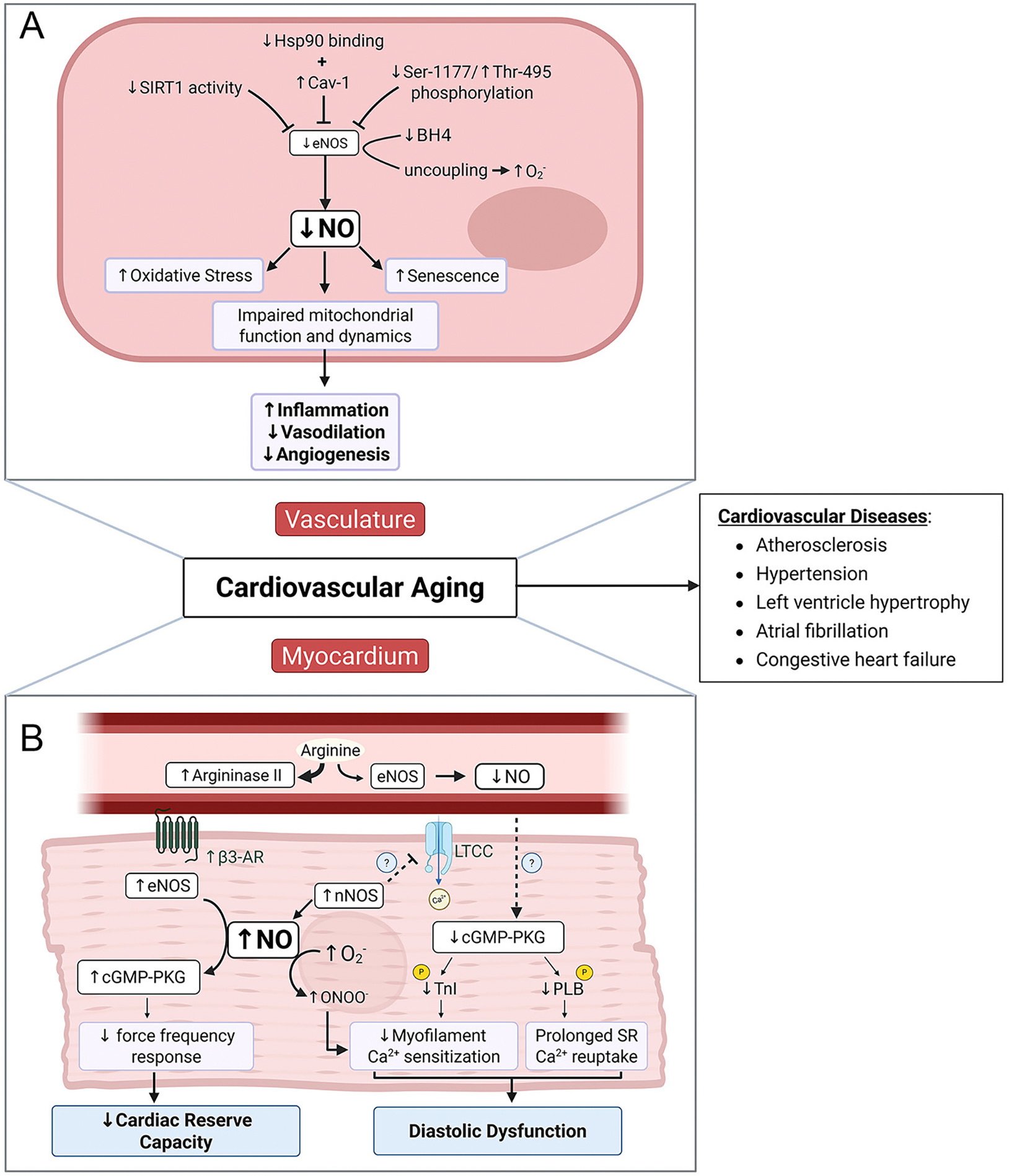
Mechanisms of Age-Associated Decline in NO Signaling and Its Contribution to Cardiovascular Dysfunction. [(A): Vasculature] In the vascular endothelium, aging-associated declines in eNOS activity and NO bioavailability are driven by reduced SIRT1 activity, elevated Cav-1 expression, and diminished Hsp90-eNOS interaction. Post-translational modifications - specifically, reduced phosphorylation at Ser1177 and increased phosphorylation at Thr495 - further suppress eNOS activity. Concurrently, age-related depletion of BH_4_ levels promotes eNOS uncoupling, shifting its activity toward O_2_^−^ production. The resulting reduction in NO bioavailability contributes to increased oxidative stress, mitochondrial dysfunction, and endothelial cell senescence. Collectively, these changes impair vascular function by promoting inflammation, reducing vasodilation, and attenuating angiogenesis. [(B) Myocardium] With advancing age, diminished NO bioavailability in the endothelium reduces paracrine signaling to cardiomyocytes, likely contributing to the downregulation of the cGMP-PKG pathway and, ultimately, diastolic dysfunction. This decline is further aggravated by the formation of peroxynitrite, generated from the interaction between nNOS-derived NO and O_2_^−^. In contrast, heightened β3-adrenergic receptor activity stimulates cardiomyocyte-associated eNOS-cGMP-PKG signaling, which leads to reduced inotropy, particularly at elevated heart rates, thus lowering cardiac reserve capacity. Plasmalemmal translocation of nNOS may further act to inhibit L-type calcium channels and negatively impact cardiac EC coupling, although this remains to be fully established. Together, age-associated declines in NO bioavailability in both the vascular endothelium and myocardium contribute to cardiovascular dysfunction and elevate the risk of life-threatening cardiovascular diseases. Created in BioRender. Patel, P. (2025) https://BioRender.com/v5hq0f8.

In summary, age-related changes in NO signaling are multifactorial and affect vascular health through complex and interconnected mechanisms involving altered NOS expression and activity, cofactor availability, increased oxidative stress, and disrupted mitochondrial metabolism. These disruptions contribute to hallmark features of vascular aging, including endothelial dysfunction, arterial stiffness, and impaired metabolic resilience [Figure 2]. Critically, the processes of vasodilation, oxidative stress, metabolism, cellular senescence, inflammation and angiogenesis are not distinct processes but rather are interconnected dynamically, with reduced NO availability acting as a central link. Reduced NO availability limits vasodilatory capacity and promotes oxidative stress, which in turn amplifies inflammatory signaling and accelerates endothelial dysfunction. These changes further reduce NO availability which stimulates cellular senescence, further impairing NO signaling and metabolic flexibility within the vascular wall. Viewing these mechanisms as interconnected nodes of a dynamic system highlights the crucial role of NO in cardiovascular aging and its potential as a target to decelerate cardiovascular aging. Given the central role of NO in cardiovascular physiology, many of these mechanisms may also extend beyond the vasculature to influence myocardial aging - a topic explored in the next section.

## NO AND MYOCARDIAL AGING

In conjunction with vascular impairment, normal cardiovascular aging is associated with negative alterations in myocardial structure and function, including impaired diastolic filling, reduced cardiac reserve capacity, left ventricular (LV) hypertrophy, atrial enlargement, and changes in cardiac rhythm or rate^[161]^. While these changes do not constitute overt cardiovascular disease, they significantly reduce exercise tolerance, increase frailty, and lower the threshold for clinical symptoms, ultimately diminishing quality of life. Moreover, age-related cardiac remodeling alters the myocardial substrate in ways that amplify the severity of heart disease and contribute to poorer outcomes in older adults. Indeed, among all comorbidities, aging-related cardiovascular dysfunction is a key contributor to the disproportionately higher incidence of LV hypertrophy, atrial fibrillation, and congestive heart failure observed in older adults^[162–164]^, underscoring the need to identify underlying molecular mechanisms and potential therapeutic targets.

### Changes in NO bioavailability in myocardial aging

Although numerous studies have examined age-related changes in NOS expression and activity in the aging myocardium, conclusions regarding NO bioavailability and the specific origins of NOS-associated effects - whether from the coronary endothelium or cardiomyocytes - remain discrepant and incomplete. In general, nNOS expression within the heart has been shown to increase with age^[165–167]^, whereas eNOS has been reported to be unaltered^[100,167]^, elevated^[168]^, or reduced^[166,169]^. For those few studies that have, in turn, evaluated calcium-dependent NOS activity in the aged left ventricle, most suggest an overall increase^[166,168]^. However, increased NOS activity does not necessarily equate to greater NO production or signaling. Indeed, several studies report an age-associated upregulation of Arg-II^[100,170]^, which competes with NOS for the shared substrate L-arginine, potentially limiting NO synthesis and reducing its bioavailability.

Supporting this mechanism, pharmacological inhibition of Arg-II has been shown to enhance myocardial NO bioavailability in aged rat hearts and in isolated cardiomyocytes from senescent hearts Arg-II inhibition restored the force-frequency relationship^[100]^ - a biophysical property known to be regulated by NO^[171,172]^. Similarly, L-arginine supplementation improves diastolic function in aged rat hearts^[168]^, although its direct effects on myocardial NO levels were not assessed. Beyond limitations in NO synthesis, aging is also associated with increased oxidative stress, which further depletes NO. As earlier noted, elevated O_2_^−^ levels - driven by upregulated iNOS^[100]^ and enhanced NADPH oxidase activity^[167]^ - can react with NO to form the more reactive and cytotoxic free radical, peroxynitrite^[167,173]^, leading to reduced NO signaling. Thus, while the precise effects of aging on myocardial nitroso-redox signaling remain unresolved, mounting evidence suggests that altered NOS activity, substrate competition, and oxidative stress collectively reduce NO bioavailability in the aging heart.

### Functional consequences of reduced NO in the aging myocardium

#### Calcium handling and diastolic dysfunction

Age-associated diastolic dysfunction and diminished contractile reserve are hallmark features of myocardial aging that limit cardiac performance, particularly during exertion. These functional impairments are driven by delayed calcium transient decay rates, reduced contraction amplitude, and prolonged time to peak contraction^[174–176]^. Mechanistically, these effects stem from reduced peak L-type calcium currents, sarcoplasmic reticulum (SR) calcium uptake rates, and slower cross-bridge cycling, which occur independent of broad changes in the expression of EC coupling proteins, with the exception of consistently reported reductions in sarco (endo) plasmic reticulum calcium ATPase (SERCA) expression and activity^[174,177,178]^. Rather, emerging evidence suggests that these functional deficits occur secondary to age-associated alterations in cellular signaling and impaired post-translational regulation of core EC coupling proteins.

Both autocrine and paracrine NO signaling are essential for tuning EC coupling at baseline and in response to stress^[12]^. Yet, while few studies have explored whether changes in NO bioavailability or the broader nitroso-redox balance feed into age-related cardiac dysfunction, there is reason to believe this may play a prominent role. Indeed, NO is known to modulate SR calcium uptake and, thus, cardiac relaxation via both paracrine signaling from endothelial-derived eNOS^[179–181]^ and autocrine effects of nNOS-derived NO within cardiomyocytes^[182–184]^. As mentioned above, studies using L-arginine supplementation or Arg-II inhibition have shown improvements in diastolic dysfunction in aged rat hearts owing to restored NOS-derived NO production^[100,168]^. Although speculative, cursory evidence suggests this reduction in NO bioavailability is driven by a reduction in eNOS-derived NO from the microcirculatory endothelium within the heart. This reduction appears to be linked to Arg-II upregulation^[100,170]^, which competes with NOS for the shared substrate L-arginine^[185]^, thus limiting NO synthesis. As this Arg-II upregulation is present in non-myocytic cells within the heart^[170]^, it suggests the loss of NO occurs outside of the cardiomyocyte and, thus, contributes to cardiac dysfunction via diminished paracrine NO signaling. Such paracrine NO-dependent regulation of relaxation is reported to occur through eNOS-cGMP-PKG signaling, which increases phospholamban (PLB) phosphorylation at Ser17, leading to enhanced SERCA activity and increased SR calcium uptake^[40,186]^. Studies have also shown that eNOS-cGMP-PKG signaling can directly alter troponin I phosphorylation and cross-bridge cycling kinetics, thus speeding contraction and relaxation, especially at higher heart rates^[40,180,181]^. Age-associated loss of such eNOS-cGMP-PKG signaling would, therefore, be expected to not only contribute to impaired relaxation but also limit the cardiac reserve capacity by diminishing the force-frequency relationship. Consistent with this, Arg-II inhibition not only improves NO bioavailability but also restores the force-frequency relationship in aged myocardium^[100]^.

While reductions in eNOS-derived NO may contribute to impaired vascular-cardiac signaling and myocardial relaxation with age, increasing evidence suggests that alterations in cardiomyocyte-intrinsic nNOS activity could also play a critical yet complex role in diastolic dysfunction. In young hearts, nNOS-derived NO supports efficient calcium handling and relaxation by promoting PLB phosphorylation and enhancing SR calcium reuptake via cAMP-dependent protein kinase A (PKA)^[182,183,187]^. In contrast, aging is associated with heightened nNOS activity in the context of increased oxidative stress, leading to elevated peroxynitrite formation and protein nitration. These redox-driven changes impair SR calcium cycling and increase myofilament stiffness, thereby exacerbating diastolic dysfunction^[167,173]^. Notably, genetic ablation of nNOS in aged mice reverses calcium transient prolongation and improves myocardial relaxation - an effect attributed to reduced O_2_^−^ and peroxynitrite levels - highlighting a detrimental shift in nNOS signaling with age^[167]^. These contrasting findings underscore the need for greater spatial and temporal resolution in assessing NOS isoform activity and redox dynamics in the aging heart. Additionally, age-related disruptions in NO-mediated regulation of nearby oxidases (e.g., Xanthine Oxidoreductase) and their downstream targets^[3,12]^ remain poorly defined. Thus, although altered NO signaling is a central contributor to diastolic dysfunction in the aged heart, the molecular mechanisms remain incompletely understood and may very well be context dependent.

#### Beta-adrenergic stimulation and cardiac reserve capacity

While persistent changes in EC coupling machinery are likely to diminish the inotropic capacity of the heart over time, much of the reduced cardiac reserve capacity noted in aging humans has been ascribed to attenuated beta-adrenergic responsiveness^[188–190]^, despite elevated levels of circulating catecholamines^[191]^. In senescent hearts from rats and mice, beta1-adrenergic receptor (β1-AR) expression has consistently been shown to be reduced, leading to a decrease in cAMP/PKA-dependent signaling and a diminished inotropic response^[165,188–190]^. However, in parallel, beta3-adrenergic receptor (β3-AR) expression is reportedly upregulated in the aging heart, where it contributes significantly to the attenuated inotropic response to β-adrenergic stimulation observed in both isolated muscle preparations and intact aging animals^[165]^. Both L-NAME and N5-(1-Imino-3-butenyl)-l-ornithine (L-VNIO), two pan-NOS inhibitors, partially restored the positive inotropic effect of β-adrenergic stimulation in isolated LV papillary muscles from aged hearts, indicating the involvement of NOS-derived NO. Nevertheless, it remains unclear whether the functional effects are primarily driven by eNOS-dependent coupling with β3-AR, or by nNOS relocalization to the plasmalemmal membrane, as seen in senescent post-myocardial infarction hearts^[169]^. Support for the former comes from studies in healthy, young hearts, where eNOS is known to mediate β-adrenergic signaling through both autocrine and paracrine effects of NO^[171,192,193]^. In these models, NO from eNOS has been shown to attenuate the inotropic response to adrenergic stimulation, largely through the β3-AR-NOS signaling axis^[194–196]^. This pathway involves NO’s inhibition of the L-type Ca^2+^ current and phosphorylation of cardiac troponin I, which suppresses myocardial contraction^[193,197]^. Conversely, findings in isolated ventricular cardiomyocytes suggest that nNOS-derived NO may also contribute to blunted β-adrenergic responsiveness^[187,198,199]^, specifically through its association with sarcoplasmic Cav-3^[169,198]^. Notably, sarcolemmal translocation of nNOS would not only provide a mechanistic basis for the age-related attenuation of β-adrenergic responsiveness but could also contribute to reduced myofilament calcium sensitivity and impaired force-frequency relationships, potentially explaining the decline in early diastolic filling observed in aging hearts^[3,12,200]^. While further research is needed to delineate the dominant mechanism, the interplay between NOS isoforms and β-adrenergic signaling appears likely to contribute to impaired contractile function and reduced cardiac reserve with age.

While beyond the scope of this review, it is worth noting that the development and severity of several age-associated cardiovascular diseases, including atrial fibrillation and heart failure, are strongly correlated with the extent of myocardial NOS-derived NO production (see^[12,201,202]^ for further details). Moreover, studies using nNOS knockout mice have demonstrated that loss of nNOS-derived NO alone is sufficient to increase AF susceptibility^[203]^ and promote age-dependent maladaptive LV hypertrophy, even in the absence of arterial hypertension^[171,204,205]^. Additionally, dysregulated NO signaling has been implicated in impaired lusitropy (as detailed above), mitochondrial dysfunction, and adverse extracellular matrix remodeling^[206,207]^ - key factors that exacerbate myocardial stiffness and diastolic dysfunction in aging hearts. Thus, altered NO bioavailability may not merely contribute to age-associated cardiac dysfunction but also fundamentally reshape the myocardial substrate on which heart disease is superimposed, likely worsening pathology and clinical outcomes for older patients.

## ENHANCEMENT OF NO TO PROMOTE HEALTHY CARDIOVASCULAR AGING

Across a range of model organisms, studies aimed at uncovering the molecular basis of healthy aging and extended lifespan have repeatedly converged on a common mechanism: enhanced NO signaling. From yeast to nematodes to mammals, boosting NO levels - whether through increased enzymatic production or exogenous sources such as commensal bacteria - has been linked to improved mitochondrial function, stress resistance, and longevity^[208–210]^. These findings, though diverse in model and mechanism, underscore the conserved and fundamental role of NO in promoting organismal health across the lifespan. Building on this foundation, the following section explores strategies to enhance NO bioavailability and promote cardiovascular health in humans. These include both endogenous and exogenous approaches, such as dietary nitrate supplementation, stimulation of NOS activity, and lifestyle interventions such as caloric restriction (CR) and exercise. By examining these interventions, we highlight promising approaches to counteract cardiovascular aging through targeted enhancement of NO signaling, with the hope of encouraging further testing of similar therapeutic strategies to improve longevity and quality of life in the future.

### Dietary supplementation to enhance nitrate-nitrite

As previously noted, an alternate method for nitrate and nitrite to enter circulation is through the consumption of nitrate-rich foods that are reduced by commensal oral bacteria^[48,49]^. This results in increased nitrite concentration in the saliva, some of which is then absorbed into circulation^[211]^. Following this accumulation, nitrite is physiologically reduced into NO by heme-containing proteins, including hemoglobin^[68]^. Elevating circulating nitrite levels has, in turn, been shown to improve endothelial function and reduce oxidative stress markers in healthy, older patients and aged animals^[65,212–219]^. These studies indicate that supplementation of exogenous NO sources might counteract the decreasing NO bioavailability with age. Given these results, providing nitrate/nitrite-based therapies to increase NO bioavailability for aging populations has been appealing, especially for those at risk of disease.

Beetroot juice is rich in nitrate and has been a promising, low-cost intervention strategy to reduce hypertension and subsequent cardiovascular disease risk^[220–222]^. In a recent randomized, controlled trial, the effects of beetroot juice on oxidative stress and inflammation were assessed in patients between 56–71 years old with diagnosed hypertension. Four weeks of beetroot juice mildly improved antioxidant levels and reduced some inflammatory markers^[223]^; however, consumption of beetroot juice for four weeks did not significantly alter vascular function or blood pressure in older hypertensive patients^[224]^. These results have been observed by other groups as well, although some studies do report changes in blood pressure following nitrate consumption^[225–228]^. Co-morbidities may be a key factor to consider with nitrate/nitrite therapeutics. Age and sex discrepancies, for example, may be important attributes for the effect of these therapeutics^[121,229]^. Nevertheless, nitrate/nitrite therapeutics are likely to improve age-related vascular function in healthy populations, but co-morbidities may mitigate these effects, perhaps by potential differences in NO production mechanisms^[229]^.

### Dietary supplementation to boost NOS-derived NO

Apart from dietary supplementation, interventions have also focused on increasing NO bioavailability through the stimulation of NOS production or reduction of NO scavenging by ROS. Increasing BH_4_ availability has been shown by multiple clinical studies to improve endothelial function, especially endothelium-dependent vasodilation, via increased NOS coupling and NO production^[119,230–233]^. Interestingly, however, acute BH_4_ supplementation was found to have no effect on improving vascular dysfunction in subjects older than 65^[234]^.

With age, L-arginine levels are reportedly reduced, so ingestion of L-citrulline, a precursor of L-arginine in the *de novo* arginine synthesis pathway, may stimulate endogenous de novo L-arginine synthesis. A study found that in the fasted state, patients > 60 years old with heart failure had significantly lower *de novo* arginine synthesis and NO synthesis rates compared to adults between 21–40, and citrulline ingestion resulted in an increase in NO synthesis in both groups^[235]^. While some studies have shown that L-arginine supplementation can be beneficial due to the high activity levels of arginase in the small intestine, nitrate/nitrite is considered a more effective method of increasing NO bioavailability^[218]^. However, many studies examining the effects of supplements on enhancing NOS-mediated NO bioavailability have been conducted on healthy populations, and further research is needed in populations with co-morbidities.

### Caloric restriction

CR, defined as reduced caloric intake without malnutrition or starvation, has recently garnered recognition as a promising non-pharmacological intervention to mitigate age-associated diseases and promote longevity^[236–238]^. Evidence from mice, rats, and humans subjected to varying degrees of CR has collectively demonstrated that CR alleviates age-induced increases in blood pressure^[239,240]^, lowers pulse wave velocity^[239]^, improves arterial dilation^[241]^, and reduces metabolic syndrome score^[240]^ (an indicator of cardiometabolic risk). These effects are mediated, in part, through the impact of CR on NO-dependent pathways. Indeed, CR has been shown to upregulate eNOS expression, which then promotes mitochondrial biogenesis^[242]^ and improves endothelial function in the aging vascular systems^[242,243]^. CR also supports LV function recovery in aged hearts following ischemia/reperfusion^[244]^. These beneficial effects of CR are significantly attenuated by genetic ablation or pharmacological inhibition of NOS expression^[242,243,245,246]^, thus highlighting the necessity of NOS enzymes in CR-mediated cardioprotection.

The timing of CR initiation is also a critical determinant of its efficacy, as late-onset short-term CR in older mice shows no influence on eNOS expression^[76,247]^. Interestingly, however, late-onset CR decreases iNOS expression, subsequently reducing NO levels^[76]^. While reduced iNOS expression indicates a less inflammatory state, a low NO level implies that NO replenishment might be limited with late-onset CR. Conversely, Rippe *et al.* (2010) demonstrated that short-term CR in older mice led to a slight increase in eNOS expression and a significant increase in NO bioavailability. Interestingly, this increase in NO bioavailability was primarily attributed to the CR-dependent decrease in O_2_^−^ bioactivity^[241]^. Although consensus exists regarding the involvement of NOS enzymes in CR-mediated cardioprotection, the precise duration of CR and the downstream molecular mechanisms affecting NOS activity remain to be fully elucidated.

Much of the research on CR-induced changes in NO highlights the role of SIRT1 as a key regulatory factor. Increased expression and nuclear localization of SIRT1 have been identified as pivotal upstream mechanisms driving CR-dependent increase in NOS expression and activity^[243,246]^. Deacetylation of eNOS by SIRT1 increases eNOS activity and thus, NO bioavailability^[85]^. Interestingly, elevated eNOS expression can also facilitate nuclear localization of SIRT1^[246]^, suggesting that eNOS and SIRT1 engage in a positive feedback loop to mutually regulate each other’s activity. Further investigation into the SIRT1-NOS interaction under CR is warranted to unravel the molecular basis of CR-induced vascular and metabolic improvements. Such insights could also reveal novel therapeutic targets in the CR-SIRT1-NOS axis to combat age-associated diseases and promote healthy aging.

In addition to SIRT1, several other nutrient-signaling pathways have also been implicated in modulating NOS activity under CR. For example, activation of insulin signaling, marked by activating phosphorylation of Akt, has been associated with increased adiponectin levels in serum from CR-subjected rats. This signaling cascade promotes eNOS phosphorylation, thereby enhancing eNOS activity and elevating NO production^[248]^. Moreover, CR reduces mTOR phosphorylation, rendering it inactive^[239]^, while simultaneously increasing adenosine monophosphate-activated protein kinase (AMPK) phosphorylation^[244]^. This dual modulation of the two nutrient sensing pathways may help improve cardiovascular function and prevent age-associated decline, potentially through an interaction between these nutrient sensors and NO production pathways discussed above.

While significant progress has been made in understanding the role of NO-dependent pathways in CR-mediated benefits, many critical questions remain unanswered. For instance, although CR-mediated changes in NO levels in aging vascular systems are extensively studied, the precise molecular mechanisms underlying the interplay between NOS enzymes and nutrient-sensing pathways in response to CR require further exploration. Furthermore, while the role of NO in vascular health is well-established, its role in the myocardium is less clear. Whether CR-driven improvements in myocardial function following age-related cardiac disease, such as ischemia/reperfusion^[244]^, are mediated by alterations in the NO signaling pathway remains largely unexplored. Addressing these gaps in knowledge is essential for advancing our understanding of how CR-driven modulation of NO signaling pathways contributes to protecting the aging vascular system.

### Exercise

Along with diet, physical activity is a key modifiable factor influencing cardiovascular disease risk and may exert protective effects in part by improving endothelial function and vascular health with aging^[249,250]^. Several studies have shown that sedentary individuals are more likely to develop cardiovascular diseases, whereas increased physical activity significantly reduces this risk^[251]^. Notably, NO bioavailability is lower in sedentary adults as compared to their physically active counterparts^[226]^. Recent evidence further demonstrates that older individuals who consistently engaged in more than five hours of high-intensity exercise per week over three decades had higher circulating NO levels than those who performed less than two hours of moderate-intensity exercise per week over the same period^[252]^. In contrast to the long-term effects of regular exercise, acute bouts of exercise have been shown to transiently increase NO production and reduce resting blood pressure in elderly women, while also improving vascular NO responsiveness^[253–256]^.

In aging animal models, eNOS protein expression increases despite unchanged eNOS messenger RNA (mRNA) levels; however, exercise elevates both mRNA and protein expression, leading to improved NO-mediated vasodilation and reduced arterial stiffness^[257,258]^. Enhancing NO bioavailability with age is thought not only to support vascular function but also to enhance exercise tolerance and performance in older adults^[259]^. A systematic review evaluated the effects of nitrite supplementation on exercise performance in both young and older individuals. A meta-analysis of over 100 studies and reviews revealed that while outcomes varied based on biological factors, the most substantial benefits were observed in young, healthy, and physically active men^[260]^. Thus, while the cardiovascular benefits of exercise are well established, emerging evidence suggests that increased NO bioavailability may be a key mediator of these effects^[261]^.

### Current therapeutics and future strategies

Apart from lifestyle interventions, many advances have been made towards enhancing NO bioavailability in the aging cardiovascular system. Pharmacological agents, such as sodium nitroprusside, act as direct NO donors and have historically been used for the acute treatment of hypertensive emergencies^[262]^. More recently, their safety and efficacy have also been demonstrated in clinical trials involving patients with acute heart failure^[263]^. On the other hand, nitrate esters such as nitroglycerin, isosorbide dinitrate, and isosorbide mononitrate, which are metabolized to liberate NO, are widely used for treating angina^[264]^, heart failure^[265]^, and other acute coronary syndromes^[266]^. Interestingly, however, the use of nitroglycerin in conjunction with adjunctive therapies, including antithrombotic or lipid-lowering agents, has been associated with an increased incidence of major adverse cardiovascular events in patients over the age of 75, while no measurable benefit or harm was observed in younger patients^[267]^. Similarly, a recent preclinical study in rat models found no cardioprotective benefit of nitroglycerin administration in limiting infarct size, either at baseline or in the context of metabolic syndrome^[268]^. In contrast, another study demonstrated that nitroglycerin, but not isosorbide dinitrate, improved post-ischemia/reperfusion outcomes^[269]^. These variable responses to traditional direct NO donors may stem from common co-morbidities in older patients, the lack of tissue- or pathway-specific targeting, and the heightened oxidative stress in the aging cardiovascular system, where NO rapidly reacts with O_2_^−^ to form peroxynitrite. Collectively, these limitations underscore the imminent need to better understand how NO-mediated pathways are regulated in different biological and clinical settings, and to develop more targeted, personalized therapeutic strategies that safely and efficiently elicit NO-dependent benefits in age-associated cardiovascular diseases.

Recent insights into the SIRT1-NOS-NO pathway have created such opportunities for targeted therapeutics. SIRT1-activating compounds such as resveratrol^[270]^ have garnered extensive interest as potential cardioprotective agents^[271,272]^, given that aging is associated with reduced SIRT1 activity, lower NO levels, and increased NOS uncoupling. Notably, preclinical studies have demonstrated resveratrol’s ability to reduce NOS uncoupling, enhance NO production, and limit O_2_^−^ accumulation through upregulation of guanosine triphosphate (GTP) cyclohydrolase, the rate-limiting enzyme in the BH_4_ synthesis pathway^[273]^. Clinical trials have further shown promising outcomes of resveratrol in diabetes^[274]^, ischemia/reperfusion^[275]^, hypertension^[276,277]^, and related cardiovascular diseases^[278]^. More recently, novel SIRT1 activators, such as SRT2104, have entered clinical trials with growing evidence showing their efficacy in treating cardiovascular diseases^[279]^.

Nicotinamide adenine dinucleotide (NAD^+^) serves as a key co-substrate for SIRT1. Thus, studies have also focused on NAD^+^-boosting approaches to increase SIRT1 activity. NAD^+^ precursors, such as niacin and nicotinamide riboside, have been explored for their potential to enhance SIRT1 activity and support NO signaling^[88,280]^. Preclinical studies have demonstrated their cardioprotective effects in the context of aortic aneurysms, ischemia/reperfusion, and other aging-associated cardiovascular complications^[281,282]^.

Altogether, these advances highlight the therapeutic potential of selectively enhancing SIRT1 signaling to preserve NO bioavailability in the aging heart. More broadly, these advances emphasize the promise of precision therapeutics that modulate NO signaling through pathway-specific mechanisms.

Beyond SIRT1 agonists, other classes of drugs such as peroxisome proliferator-activated receptor-alpha (PPAR) agonists^[283,284]^ and β-adrenergic receptor blockers^[285]^, are currently in clinical use and have been reported to enhance NOS activity; however, the specific pathway underlying this increase and the extent to which they contribute to NO-dependent benefits in patients with age-associated cardiovascular diseases remain unclear. A deeper focus on the upstream substrates, enzymes, and cofactors that govern NO bioavailability will be essential for ensuring consistent, clinically meaningful NO-dependent benefits in aging cardiovascular diseases.

## CONCLUSION

NO is a critical regulator of cardiovascular homeostasis, and its dysregulation represents a hallmark of vascular and myocardial aging. This review has outlined how both NOS-dependent and nitrate-nitrite pathways of NO synthesis are altered with age, contributing to a spectrum of deleterious effects - from impaired vasodilation and increased oxidative stress to reduced myocardial performance and diminished cardiac reserve. These age-associated changes in NO signaling are mechanistically linked to endothelial dysfunction, cellular senescence, inflammation, and metabolic decline, core features of cardiovascular aging. Moreover, compelling evidence across model organisms and human studies suggests that restoring NO bioavailability can counteract these effects and promote healthier aging. Interventions such as dietary nitrate supplementation, NOS-boosting nutrients, CR, and regular exercise offer promising strategies for reactivating endogenous NO pathways.

Emerging evidence highlights several promising targets for preserving NO bioavailability and mitigating cardiovascular aging. These include enzymes regulating substrate and cofactor availability (e.g., arginase II, NAD^+^ metabolism), signaling pathways such as SIRT1 that maintain eNOS activity, and mitochondrial sources of oxidative stress that contribute to NO depletion. Strategies to modulate these targets, in parallel with lifestyle interventions, represent important avenues for future translational research. Further mechanistic studies are needed to determine how these pathways interact and whether their combined modulation can enhance resilience of the aging cardiovascular system. Ultimately, targeting NO pathways represents a promising and increasingly actionable approach to extending cardiovascular health span to reduce the burden of age-related cardiovascular disease.

## Data Availability

Not applicable.
